# It shouldn’t take a murder: A call to action against sexual violence in healthcare

**DOI:** 10.1371/journal.pgph.0003929

**Published:** 2024-11-15

**Authors:** Shivangi Shankar, Abhijit Dhillon, Abhiti Gupta

**Affiliations:** 1 State Health Resource Centre, Raipur, Chhattisgarh, India; 2 Department of International Development, London School of Economics and Political Science, London, United Kingdom; 3 Independent Consultant, Kolkata, India; PLOS: Public Library of Science, UNITED STATES OF AMERICA

On August 9, 2024, a 31-year-old resident doctor at RG Kar Medical College and Hospital in Kolkata, India, was sexually assaulted and murdered on hospital premises. The news cycle picked up the case after two days, during which the hospital allegedly mishandled evidence. Initially, there were attempts to cover up the case as that of suicide, but after the mainstream media picked it up and outrage from medical residents and students followed, the case was finally declared that of rape and murder. The principal was forced to resign by protesting students, yet he was made head of another institution within hours. The institution had no mechanism to respond to such cases despite legal provisions for the same [1].

Other medical institutions in India responded with paternalistic guidelines regarding attire and timings for women doctors, later condemned by nearly everyone advocating for justice [2]. The response from the medical community is of note—the doctors’ protests have been urging for laws to protect doctors, erasing the gendered nature of the crime [2].

With the Indian Supreme Court taking Suo moto cognisance of the matter and setting up a task force for the same, such protection laws may become a reality. While it may help doctors, it lacks the nuance required to address sexual violence in the workplace and forgets that laws already exist to address sexual exploitation, abuse and harassment (SEAH) in the workplace [3].

Conspicuously missing from advocacy is representation from women, especially from outside the doctor community (the task force set up in response to the Supreme Court’s directive does not have any nursing staff), advocacy for safety across the healthcare workforce (only doctors have been talked about) and lack of accountability for the healthcare institution [4].

## SEAH in global health: What we (Don’t) know

Sadly, this situation is not unique to India. A 2022 report found that women in the health sector experience work-related SEAH that includes sexualized verbal abuse, sexual assault and rape all over the world. The report also reiterated International Labor Organization (ILO) findings of gross underreporting regarding SEAH in the workplace [5]. Most crime records, too, fail to capture the layered vulnerability across caste, race, class, gender, disability, profession, and social status. Added risks in disaster and conflict settings, where health workers are inevitably stationed, are often missing from these datasets [6].

In September 2020, reports surfaced of sexual abuse during the Ebola response from 2018 to 2020 in the Democratic Republic of Congo (DRC) revealing widespread sexual exploitation, harassment, and abuse by local leaders and staff from local, national, and international organisations—including staff within the World Health Organization. As per reports, sexual favours were asked in exchange for employment. The victims were vulnerable women from the region who ended up working as frontline sanitation or aid workers during the outbreak [7].

Women constitute 70% of the health workforce while holding just 25% of leadership roles [8]. This gendered vertical segregation in healthcare, i.e., men primarily occupying higher-level, decision-making positions of power while women work in “lower-skilled”, lower-paid and informal or “voluntary” roles excludes them from protection, justice, and accountability mechanisms [9]. This division is most acutely felt by community health workers (CHWs) and other informal healthcare workers, whose workplaces often lack clear boundaries. Consequently, millions of women CHWs, midwives, and other allied staff face a heightened risk of sexual exploitation, abuse, and harassment (SEAH), with incidents downplayed or silenced [7, 10].

## The way forward

**Fig 1:**
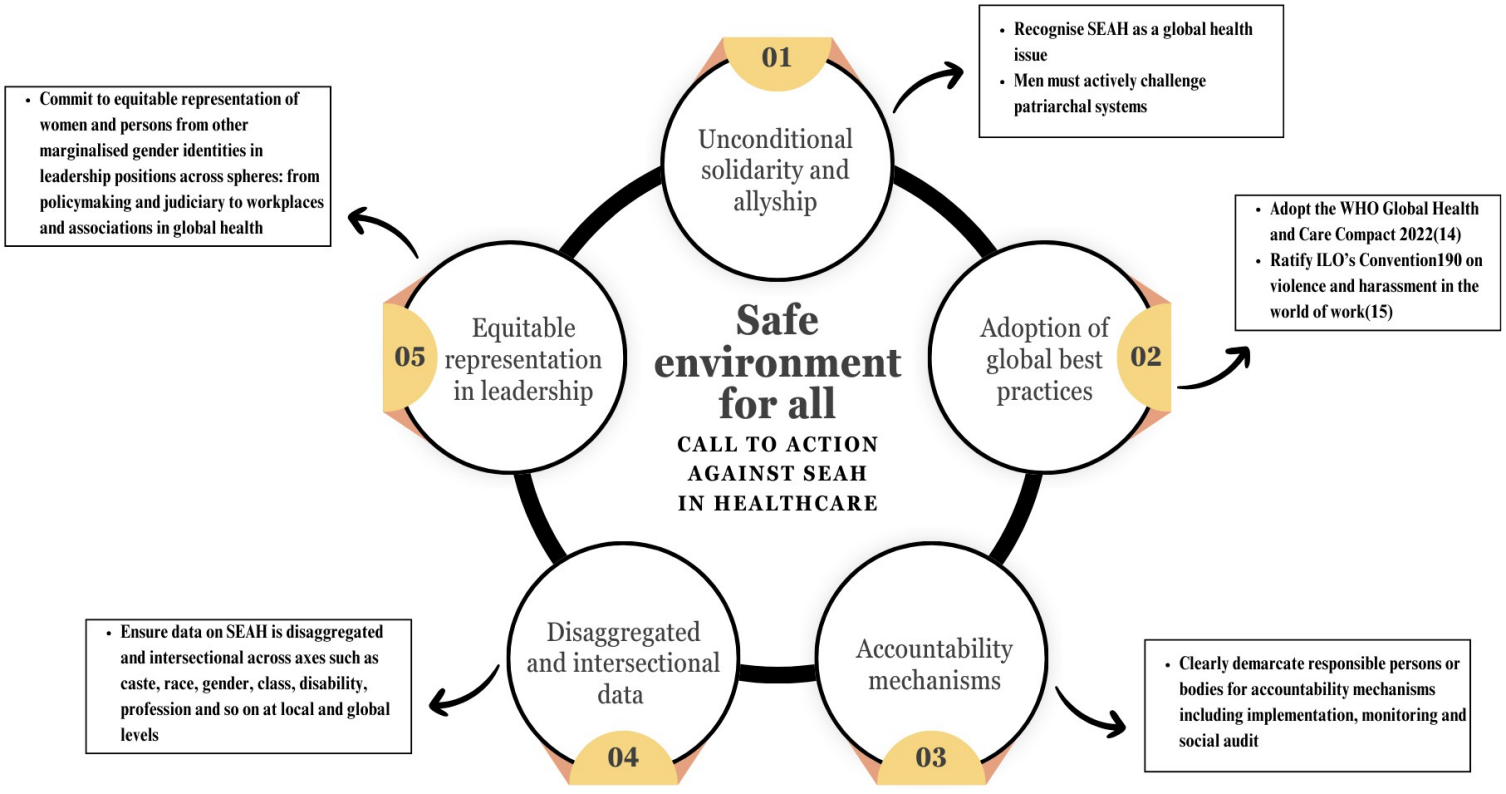
Safe environment for all- *call to action against SEAH in healthcare (Original figure)*.

Global responses to SEAH in healthcare are sporadic, isolated, and unsustainable. It shouldn’t take a murder, such as the recent events in India, for health systems to evolve into safe environments for all. Workplaces in healthcare must take responsibility to ensure safety for all who interact with it. We urge all actors within the health system to prioritize SEAH in the workplace as a global health issue and join hands for concerted action (Fig 1). Policymakers at all levels must adopt global best practices for the prevention of SEAH such as the 2022 WHO Global Health and Care Compact which sets out comprehensive actions in 4 areas: preventing harm; providing support; inclusivity and safeguarding rights [11]. We also urge for ratification of the International Labour Organization’s (ILO) Convention 190 on Violence and harassment in the world of work. As more countries ratify ILO Convention 190 which launched in 2021 and is the first global convention to address work-related violence and harassment specifically, the burden of shame and the responsibility of ensuring safety shifts away from victims and survivors to perpetrators and institutions [12].

While these have value at the policy level, we recognise the need for effective implementation across levels. Thus, we recommend setting up accountability mechanisms within workplaces which demarcate responsibility for ensuring the implementation, monitoring and social audit of SEAH prevention and response mechanisms at the institutional level. There is also a need to fill the gap between women’s experience of SEAH in the workplace and the available data regarding it, especially disaggregated and intersectional data. Yearly surveys on SEAH at the workplace designed with inclusive and diverse experiences of all within the workplace should become a norm. We also demand equitable representation of women and persons from other marginalised gender identities in leadership positions: from policymaking and judiciary to workplaces and health associations.

Men across organisations have an ethical and political responsibility to challenge and dismantle these dynamics, including addressing SEAH, in the workplace and society. We urge men to step up as allies in the fight against SEAH.

The furore around the Indian case must evolve into a sustained global demand for justice and reform. Being safe is a human right, and this human right extends to the workplace. Lawmakers, policymakers, directors, managers, and colleagues at all levels need to play a role in ensuring the prevention of SEAH in healthcare and maintaining a safe work environment for all.

## References

[1] Kumar S. IN THE SUPREME COURT OF INDIA CRIMINAL ORIGINAL JURISDICTION SMW (Crl) No 2 of 2024 IN RE: ALLEGED RAPE AND MURDER INCIDENT OF A TRAINEE DOCTOR IN R.G. KAR MEDICAL COLLEGE AND HOSPITAL, KOLKATA AND RELATED ISSUES Versus [Internet]. 2024. https://main.sci.nic.in/supremecourt/2024/37351/37351_2024_1_66_54902_Judgement_20-Aug-2024.pdf

[2] Choudhary R. NDTV. Kolkata Doctor Rape, Murder, RG Kar Medical College: Assam Hospital’s Bizarre Memo After Kolkata Shocker, Then A U-Turn;2024 https://www.ndtv.com/india-news/kolkata-doctor-rape-murder-rg-kar-medical-college-avoid-being-alone-assam-hospitals-advisory-after-kolkata-rape-murder-6333986

[3] Kumar S. IN THE SUPREME COURT OF INDIA CRIMINAL ORIGINAL JURISDICTION SMW (Crl) No 2 of 2024 IN RE: ALLEGED RAPE AND MURDER INCIDENT OF A TRAINEE DOCTOR IN R.G. KAR MEDICAL COLLEGE AND HOSPITAL, KOLKATA AND RELATED ISSUES Versus [Internet]. 2024. https://main.sci.nic.in/supremecourt/2024/37351/37351_2024_1_66_54902_Judgement_20-Aug-2024.pdf

[4] Rajgopal K. The Hindu. Kolkata rape and murder case: SC says incident final straw, forms task force to frame protocol for doctors’ safety; August 2024. https://www.thehindu.com/news/national/sc-constitutes-10-member-task-force-to-formulate-protocol-for-ensuring-safety-of-doctors/article68546044.ece

[5] Women in Global Health. Health too: Ending Sexual Violence and Harassment of Women Health Workers; 2022. Women in Global Health. https://womeningh.org/healthtoo/

[6] UN Women. Background paper: A synthesis of evidence on the collection and use of administrative data on violence against women [Internet]. UN Women—Headquarters; 2024. https://www.unwomen.org/en/digital-library/publications/2020/02/background-paper-synthesis-of-evidence-on-collection-and-use-of-administrative-data-on-vaw

[7] Flummerfelt R Ange. EXCLUSIVE-New sex abuse claims against aid workers exposed in Congo [Internet]. Reuters; 2021. https://www.reuters.com/article/business/healthcare-pharmaceuticals/exclusive-new-sex-abuse-claims-against-aid-workers-exposed-in-congo-idUSL8N2L953V/

[8] Women in Global Health. Policy Brief: The State of Women and Leadership in Global Health. Women in Global Health; 2023. https://womeningh.org/sheshapes/

[9] Sreerekha MS. State Without Honour. Oxford Academic; 2017. https://academic.oup.com/book/3610

[10] Rao L, Prakash R, Rai P, Tharakan M, DL K, Kar A, et al. Investigating violence against Accredited Social Health Activists (ASHAs): a mixed methods study from rural North Karnataka, India. Journal of Global Health Reports; 2021. https://www.joghr.org/article/24351-investigating-violence-against-_accredited-social-health-activists_-ashas-a-mixed-methods-study-from-rural-north-karnataka-india

[11] World Health Organization. Global health and care worker compact: technical guidance compilation. [Internet]. Geneva: World Health Organization; 2022. https://www.who.int/publications/i/item/9789240073852

[12] International Labour Organization. Violence and Harassment Convention, 2019 (No. 190). [Internet]. Geneva: International Labour Organization; 2019. https://normlex.ilo.org/dyn/normlex/en/f?p=NORMLEXPUB%3A12100%3A0%3A%3ANO%3A%3AP12100_ILO_CODE%3AC190

